# A comparative ethical analysis of the Egyptian clinical research law

**DOI:** 10.1186/s12910-024-01040-0

**Published:** 2024-04-30

**Authors:** Sylvia Martin, Mirko Ancillotti, Santa Slokenberga, Amal Matar

**Affiliations:** 1 Center for Research and Bioethics, Uppsala University, Uppsala, Sweden; 2 Department of Law, Uppsala University, Uppsala, Sweden; 3 Clinical Immunology and Transfusion Medicine Department, Uppsala University Hospital, Uppsala, Sweden

**Keywords:** Biomedical laws, Ethical principles, Egypt, Clinical trials, Social value

## Abstract

**Background:**

In this study, we examined the ethical implications of Egypt’s new clinical trial law, employing the ethical framework proposed by Emanuel et al. and comparing it to various national and supranational laws. This analysis is crucial as Egypt, considered a high-growth pharmaceutical market, has become an attractive location for clinical trials, offering insights into the ethical implementation of bioethical regulations in a large population country with a robust healthcare infrastructure and predominantly treatment-naïve patients.

**Methods:**

We conducted a comparative analysis of Egyptian law with regulations from Sweden and France, including the EU Clinical Trials Regulation, considering ethical human subject research criteria, and used a directed approach to qualitative content analysis to examine the laws and regulations. This study involved extensive peer scrutiny, frequent debriefing sessions, and collaboration with legal experts with relevant international legal expertise to ensure rigorous analysis and interpretation of the laws.

**Results:**

On the rating of the seven different principles (social and scientific values, scientific validity, fair selection of participants, risk-benefit ratio, independent review, informed consent and respect for participants) Egypt, France, and EU regulations had comparable scores. Specific principles (Social Value, Scientific Value, and Fair selection of participants) were challenging to directly identify due to certain regulations embodying 'implicit' principles more than explicitly stated ones.

**Conclusion:**

The analysis underscores Egypt's alignment with internationally recognized ethical principles, as outlined by Emanuel et al., through its comparison with French, Swedish, and EU regulations, emphasizing the critical need for Egypt to continuously refine its ethical regulations to safeguard participant protection and research integrity. Key issues identified include the necessity to clarify and standardize the concept of social value in research, alongside concerns regarding the expertise and impartiality of ethical review boards, pointing towards a broader agenda for enhancing research ethics in Egypt and beyond.

**Supplementary Information:**

The online version contains supplementary material available at 10.1186/s12910-024-01040-0.

## Introduction

Science relies on research to move forward and enhance knowledge. Different research areas deploy at different levels of human subjects’ involvement, from qualitative and non-interventional research methods to biomedical research and medical validation procedures. While virtually all research has ethical implications, clinical research calls for special attention, and it is widely agreed that it should be conducted according to more stringent ethical principles than other types of research [1]. To facilitate appropriate ethical implementation of research, it is imperative for ethical regulations to be receptive to the advancements in the field. The well-being of participants is a fundamental condition of research emphasized by the principles outlined worldwide in renowned ethical texts like the Belmont Report (cited in [2]^1^), and the Helsinki Declaration (see [3]^2^). These core principles include ensuring participants' entitlement to minimize harm and discomfort [2], as well as safeguarding their rights against exploitation [4].

For assessing whether the ethical requirements are fulfilled, and to ensure that international standards are respected, as it has been proposed by Artal & Rubenfeld [5], 2017, it is essential account for specific principles developed in the field. Research conduct that seemed legitimate to the men of science in the past is abhorrent to the contemporary conscience [6]. Ethical standards also depend on where they apply. Different societies, with their specific traditions and cultures, have systems of values and norms that may only partly coincide with research ethics principles informing international standards. Hence, there could be a gap between what is culturally acceptable and what is compliant with international ethics standards. However, the risk of ethical colonialism and its biases, may be difficult to avoid, as it can be considered factual that many international documents heavily rely on the Western perspective [7].

While cognizant of this, a few documents can be considered ethical reference points, such as the above-mentioned Belmont Report and the Helsinki Declaration for the protection of human participants in medical research [8]. Another influential example is the Ethical Framework for Biomedical Research from Emanuel et al. [9, 10]. The Ethical Framework for Biomedical Research has heavily influenced the ethics work of leading institutions such as the Department of Health (DoH, South Africa^3^) and Council for International Organizations of Medical Sciences (CIOMS). In research ethics, the framework has often been used to assess the functioning of ethical review committees and the ethical adequacy of legal regulations of research involving human subjects (see [11–13]). This ethical framework, rooted in major Western philosophical traditions but not explicitly aligned with any specific school of thought, enables the authors to formulate a set of principles that resonates with a broad consensus, accommodating diverse moral intuitions and beliefs.

Regulatory framework implementation in the new settings offers a great chance to explore what the most recent bioethical laws are (like for the BRICS countries in [14]). In this regard, the Egyptian Bioethical law from 2020 can be considered as an innovative example for other countries in the process of implementing bioethical regulations and improved bioethical education across the world [15–17].

As Egypt is considered an LMIC by the World Bank [18], yet a “high growth pharmaceutical market”, the country has become one of the most attractive locations for pharmaceutical companies to outsource their clinical trials. The country, with over 100 million inhabitants, provides a noteworthy example of implementing bioethical laws in a context with predominantly treatment-naive patients and a robust medical infrastructure encompassing public hospitals and healthcare professional representation.

The aim of the present paper is to analyze and discuss from an ethical perspective the new Egyptian clinical trial law. The Egyptian law is analyzed and discussed in relation to the Ethical Framework for Biomedical Research by Emanuel et al. [9, 10], and in comparison to selected other national and supranational laws.

### Egypt

One of the recent countries adopting bioethical law to regulate clinical human subject research is Egypt, which enforced its first law on clinical trials in the official journal on December 23rd, 2020. The issuance of the law, which has long been in the making, was hastened by the COVID-19 pandemic and the urgency to carry out vaccine trials among the Egyptian population [19]. This regulation is part of a broader effort to enhance the respect for civil/human rights in the country. In 2022, the recent reports from the US Embassy still pointed out some issues [20], raising concern about fairness and equity in the whole society, and impacting ethical procedures in health and research. However, Egypt -as a United Nations member since 1945- has been participating in the global initiative to enhance human rights application [21]. Like other Arabic countries (Jordan, Saudi Arabia) registered in the UN Watch Database, it needed to justify their application of human rights [22]. Efforts are made to improve ethical skills among health care givers in Egypt. For example, EL-Khadry et al. [23], assessed the effect of educational intervention on knowledge and attitude towards research, research ethics, and biobanks among Egyptian paramedical and administrative teams. Egypt has witnessed exponential growth in medical research like in many developing countries, driven by the pressing need to improve healthcare [24]. Egypt held the 37th position in terms of publication volume in 2023 [25]. It is worth noting that in 2020, Egypt had only 838 researchers per million inhabitants, in stark contrast to the USA’s 4,821 researchers per million inhabitants (in 2019) and the United Arab Emirates’ 2,443 researchers per million inhabitants (Researchers in R&D (per million people) [26] representing the medium position compared to of BRICS countries like South Africa (484 researcher per million habitants) or China (1,585).

### National examples: France and Sweden

On the national level, France and Sweden hold a long tradition of ethical regulation. French law influenced the structuring of the Egyptian legal system in 1875. Later, reforms were made to the Egyptian civil law under the guidance of a French legal expert Édouard Lambert in the 1930-the 1940s [27]. We selected France as a study focus due to its historical ties and influence on Egypt's regulatory framework. Additionally, for comparative analysis with another high-income Western nation, Sweden was chosen for its renowned status as a research leader, distinct from any historical connections with Egypt.

Northern European countries are still considered to be leading countries in research (Sweden is the 3^rd^ country in terms of research and development expenditures (% of GDP) after Israel and Korea in 2020 – [28] and have a long tradition of bioethics practices and reflections (e.g., Helsinki’s declaration in 2000 [29]). Specifically, Sweden is included in the study as an example of a Nordic country with an evidence-based culture of health policymaking [30] and constant interest for ethical inquiry in under-researched vulnerable populations [31–33]. In 2004, Sweden enforced “The Act concerning the Ethical Review of Research Involving Humans” (SFS nr: 2003:460) that sharpened ethical review procedures for biomedical research way earlier than other countries (e.g. Loi Jardé in France from 2012, and the Egyptian law 2020), introducing a reference that showcased innovative law in the early 2004 that remains in effect. In 2020, France had 4926 researchers per million inhabitants, quite comparable to the USA’s 4,821 researchers per million inhabitants (in 2019) and representing a European example of a “medium” score of researchers per million inhabitants. In comparison, Sweden counts for 7,930 researcher per a million inhabitant, Norway 6,699, Finland 7,527 or Denmark with 7,692 (Researchers in R&D (per million people) [34]. At the international level, France ranked at the 6^th^ position for publication volume, while Sweden ranked the 18^th^ [35].

### Supra-national entity: the EU regulations to consider when considering France and Sweden

Supra-national European regulations play an important role in the legal system of EU countries even if such a supra-national level does not exist in Egypt. At the EU level, the EU Regulation on clinical trials on medicinal products for human use (CTR) governs the ethical review of clinical trials, however detailed aspects of ethics committees and ethical review depend on further regulation at Member State level. This means that even though the EU regulates ethical review of clinical trials in the CTR, there could be considerable divergences across Europe in how the committees are set up and perform their tasks. To begin with, the CTR requires that a clinical trial be subject to ethical review (Article 4), and it outlines several relevant aspects of the process of carrying out that review. However, modalities regarding ethical committee and its work are a question of the Member States’ regulation. Generally, the application for authorisation to conduct a clinical trial is divided into two parts. Part I focuses on the technical-scientific dimension, and part II on the ethical aspects which are reviewed by each member state concerned. An ethics committee, within the meaning of the CTR, is an independent body established in a Member State in accordance with the law of that Member State^4^. Under the national law, this body needs to be empowered to give opinions for the purposes of the CTR, considering the views of laypersons, in particular patients or patients’ organizations (Art. 2(2)(11)). The CTR prescribes in Recital 18 merely a guiding requirement that the member state needs to ensure that “the necessary expertise is available”. Member States should have a mechanism in place to ensure the involvement of laypersons, in particular patients or patients’ organisations. However, the effect of this involvement that the CTR requires is that their views are taken into account in the review (Art. 2(2)(11)). It is not uncommon that several ethics committees exist in a member state. How the involvement of an ethics committee is organized for the purposes of the tasks specified in the CTR is a question for the Member States to decide (recital 18). However, the process needs to be organized so that the relevant timelines of the clinical trials approvals set out in the CTR are met (Art. 4).

## Methods

### Design and data analysis

We examine the Egyptian law vis-à-vis France’s and Sweden’s framework, considering the obligations that stem in regards to clinical trials from the CTR. Furthermore, we examine these regulations in light of the Ethical Framework for Biomedical Research by Emanuel et al. [9, 10]. Indeed, we will consider the EU implication into France and Sweden’s regulations.

A directed approach to qualitative content analysis was adopted using the seven principles informing the Ethical Framework for Biomedical Research as predetermined themes [36]. Two independent coders examined each selected regulation in their original version for French (MA, SM), Swedish (MA, AM), EU (MA, AM), and in an English translation for the Egyptian law (AM, SM). The coders discussed the results critically in debriefing sessions and their coding was discussed until consensus with a legal expert working with ethical regulations at the international level (SS) contributing the clarification of EU and both Swedish and French framework.

### Theoretical framework

The seven principles that will serve as comparison criteria for our analysis are the following: “*(1) (Social) value* - *enhancements of health or knowledge must be derived from the research; (2) scientific validity- the research must be methodologically rigorous; (3) fair subject selection - scientific objectives, not vulnerability or privilege, and the potential for and distribution of risks and benefits, should determine communities selected as study sites and the inclusion criteria for individual subjects; (4) favorable risk-benefit ratio-within the context of standard clinical practice and the research protocol, risks must be minimized, potential benefits enhanced, and the potential benefits to individuals and knowledge gained for society must outweigh the risks; (5) independent review - unaffiliated individuals must review the research and approve, amend, or terminate it; (6) informed consent - individuals should be informed about the research and provide their voluntary consent; and (7) respect for enrolled subjects -subjects should have their privacy protected, the opportunity to withdraw, and their well-being monitored.*” (Emanuel et al., 2000, p2701 [9]).

### Material

For a thorough assessment, we exclusively examined the primary text of each law, excluding connections to other regulations (e.g., the "Code de la Santé" and "Code Penal" for French regulation or to “Law No. 151 of 2019, the Egyptian Medicines Authority” for the Egyptian text). Our analysis utilized the latest version of the law, including any amendments. These are:

1. *Egypt’s law* – no amendments December 23^rd^, 2020, Law No. 214 of 2020 Regulating Clinical Medical Research.
2. *French law*: the “Loi Jardé” (LOI n° 2012-300 du 5 mars 2012 relative aux recherches impliquant la personne humaine) amended with the “Décret n° 2016-1537 du 16 novembre 2016 ”. We will consider the latest 2022 amendment for reference in our analysis.
3. *Swedish law*: *Lag (2003:460) om etikprövning av forskning som avser människor* with the following amendments: 2018:147, 2018:1092, 2019:1144, 2021:611, 2022:48.
4. Regulation (EU) No 536/2014 of the European Parliament and of the Council of 16 April 2014 on clinical trials on medicinal products for human use, and repealing Directive 2001/20/EC Text with EEA relevance OJ L 158, 27.5.2014, p. 1–76.

These laws have been identified for being the main relevant legal texts for biomedical research regulations and more specifically research involving human subjects. The selection of national and international regulations to assess was based on their presence within international pharmacological and biomedical research industry. Moreover, the EU regulation will be considered as an adjunct line of analysis to complement Swedish and French regulation examination as both countries are part of EU.

## Results

The full overview of coding procedure for all regulation is available as supplementary material (see Tables 1, 2 and Appendix 1). A summary of this assessment is presented in Table 1 with a score system showing the compliance or absence of compliance to each principle. A score of 0 means there is little to no compliance with the criteria, while an X indicates satisfactory or complete compliance with the criteria.

**Table 1:** Comparative coding summary

Emanuel’s criteria	Egypt	France	Sweden	EU
1 Social values and scientific value	0	0	0	0
2. Scientific validity	x	x	x	x
3. Fair selection	x	x	0	x
4. Favorable Risk-benefit ratio	x	x	x	x
5. Independent review	x	x	x	x
6. Informed consent	x	x	x	x
7. Respect for participants	x	x	x	x

0= partial or no compliance with the criteria, X=satisfactory or full compliance with the criteria

**Table 2:** Coding table short form

Emanuel et al., 2000 [9]	French Law	EU regulation	Egypt	Sweden
Social and scientific value	Article L1121-2 Modifié par LOI n°2012-300 du 5 mars 2012 - art. 1 (V) Article L1121-16	Article 3 General principle, Article 6, See art.78 and 79.	Art 10, Chapitre 5, art 18. Chapitre 8 art 20. Art 7; 2 - Ch 2 Art 10. Executive regulations art 24	No provisions..
Scientific validity	Art. L 1121-3, Article L.1121-8-1, Arti. L1123-7	Article 4 Prior authorization, Article 6	article 1, #2, art 1, #24, Art 7; 2, Ch 2 Art 10 Ch 5 art 22. Chap 3 art 6, §2 Chap 4 art 9.	11 §, Verksamhetsregioner och avdelningar av Etikprövning 25§, Beslutsförhet 26 §
Fair selection of study population	Articles L. 1121-5 to (Articles L. 1121-5 to L. 1121-8 of the Code de la santé Code – CSP. article L. 1122-2, article L. 1122-2 of the CSP, article L. 1122-1-2 of the CSP, art. L. 1122-2 II, §3, art. L.1122-2 PHC, Art. L. 1121-6 PHC, art. L.1121-8-1 PHC, art. L.1122-1-2 PHC, art. L.1122-1-3 PHC, art L.1131-1-1 PHC, art. L1131-1-1 PHC, art. L.1122-2 II PHC, art. L. 1122-2 II PHC.	Article 10 Specific considerations for vulnerable populations, Article 35, Article 31 Clinical trials on incapacitated subjects, Article 28 Article 29(2), Article 32 Clinical trials on minors, Article 33 Clinical trials on pregnant or breastfeeding women, Article 34.	Art 17; 7 (PI resp), art 3, Chap 5 art 13.14:	14 §
Favorable risk-benefit ratio	article L1123-10 (R. 1123-46),Article L1121-2)	Article 6	Art 18; 6 (PI duties), Chap 5 art 10, Chap 7 art 18 §9, Chap 11 : requirements of research organization	8 §, 9 §, 10 §
Independent review	L. 1114-1, Art. L1123-1	Article 4, Article 9	- YesArt1; 24, law art 8., Chap 2 article 4: REC, Chap 3 artile 1 to 4 about setting up protocols	6 §, 25 § SFS 2018:1091 Act with supplementary provisions on ethical review to the EU regulation on clinical trials of human medicinal products Ethical review of the application for permission for clinical drug trials § 2 The ethical review must be carried out by the Ethics Review Authority.
Informed consent	Article L1121-2, Article L1121-14, Chapter II: Informing and obtaining the consent of persons undergoing research involving the human person (Articles L1122-1 to L1122-2). Chapitre V : Dispositions particulières applicables aux investigations cliniques de dispositifs mentionnés à l'article premier du règlement (UE) 2017/745 du Parlement européen et du Conseil du 5 avril 2017 (Articles L1125-1 à L1125-31)	Article 7, Article 29, Informed consent, Article 76(1); Article 81 (the ‘EU database’), Article 37(4), Article 30 Informed consent in cluster trials	Art 1; 21, Art 12; 3 Chap.5 arti 12 § 3, law Art 3, Chap 7 art 17 § 2: obtaining IC is mandatory, Chap 10, art 23 §2 Informed consent for data usage for further research	!6 §, 14 §, 17 §, 18 §, 20 §, 21 §, 22 § All the sections in the law from 13, 14, 15, 16, 17, 18, 19, 20, 21 and 22.
Respect for recruited participants and study communities	art L 209–5,. art. L.1122-1 PHC, art. L.1122-1 HPC, art L1126-1 PHC Concerning interventional researches (category 1): art. L.1122-1-1 PHC, art. L. 1122-1-1 PHC, art. L.1122-1-1, §2, PHC.	Article 28 General rules, Article 29(2) Article 29(1), (7) and (8), Chapter 5	Art 12; 2., Art, 15; 2.Chap 5, Art 15;3, Art 12 (1), art 18:5, Art 20:9, 10, Art 14	1§, 7 §, 19 §, 8 §

### Social value

#### Egypt

Egyptian law does not overtly address the social value of the research proposal. However this may be implied as the national Research Ethics Committee (REC^5^) (Supreme Council) will take into account “national interest” when evaluating research protocols (Chapter (ch) 3, article (art) 7(2)).

#### Sweden

Similarly, the Ethical Review Act, has no clauses that are focused on assessing the social value of research. However, the notion of research serving a social interest is implicitly demonstrated by the composition of the departmental REC, where five members out of 15 represent society’s interests (Section 25). In Section 8, it is indeed stated that the welfare of research participants must be prioritized over the needs of society.

#### France

France also lacks a clear statement on the social value. The law relates to “social” level as it often refers to the “Code de la santé” (CS) and to the “social security” system, but no clear points about social values per se. Social and scientific value is stated in the “Research organized and carried out on human beings to develop biological or medical knowledge shall be authorized“ (Art. L1121-1 CS).

#### EU

Under the CTR, social value – enhancement of health – is the whole purpose, even if not expressis verbis stated, Art. 3 and Art. 6 ensure that as a general principle, (a) the rights, safety, dignity, and well-being of subjects are protected and prevail over all other interests; and (b) it is designed to generate reliable and robust data for example. Moreover, member state and Union inspections are envisaged (taking compliance with the EU regulation as a token of good research for society and for science). See Art. 78 and 79.

### Scientific validity

#### Egypt

Regarding Scientific validity, Egyptian law set up the responsibility of REC to ensure ethical quality (Art. 1, Art. 2, Art. 24) of the accepted protocols, but also set up standards for scientific quality (Art. 7; 2; Ch. 2 Art. 10) making sure that principal investigators have the required scientific competences (Ch. 5 Art. 22; Ch. 3 Art. 6 provided a detailed list of required competences §2, Ch. 4 Art. 9.).

#### Sweden

The Swedish law emphasizes the importance of sound research. In Section 11, it is stated that research may only be approved if it is carried out by/under the supervision of a researcher with the necessary scientific competence. In Section 9, where the scientific value of the proposed research is weighed against, and if proportionate justifies, the risks to the health, safety, and personal integrity of research participants.

#### France

Article L1121_2 expresses the need for social and scientific validity. Art. L 1121-3 refers to “qualified personnel” for scientific validity and after the approval has been given by a REC. RECs have a regional organization and can be involved together with *Commission nationale de l'informatique et des libertés* (CNIL) for data security issues, and Committee of Experts for Research Study and Evaluation in Health domain. Any research is also regulated by EU rules (Article L1121-1 CS). Specific regulations for certain disciplines are stated in the CS (L1121-3) but not in the Loi Jardé.

#### EU

At the EU level, Art. 4 requires prior authorization. In particular, a clinical trial shall be subject to scientific and ethical review and shall be authorized in accordance with the rules set out in the CTR. Under Article 6(1)(b)(i) the reliability and robustness of the data generated in the clinical trial, taking account of statistical approaches, design of the clinical trial and methodology, including sample size and randomization, comparator, and endpoints.

### Fair selection of study population

#### Egypt

The Egyptian law ensures the impartial selection of an appropriate number of research participants. There are specific recommendations for REC in regards to recruitment of specific sub-groups or vulnerable population. For example, prohibit research participants to enroll in simultaneous medical research and prohibit induced participation (Ch. 5 Art. 13; 14).

#### Sweden

There are no definite clauses in the considered law that emphasize the requirement for fair selection of the study population. Nonetheless, protection of minors and individuals who cannot consent to research participation is described in Sections 18, 20, 21, and 22 (see Informed consent section).

#### France

French regulation requires that study participants be beneficiaries of the Social Security system (and if not, they will be considered as if they are). Fair selection of the participants is ensured by the CS (Articles L. 1121-5 to L. 1121-8). The main categories with stated protection are adults in coma, with dementia or for psychiatric conditions, or enfeebled patient, people deprived of their freedom, foreigners, minors, pregnant and nursing women. Moreover, situations such as “urgency” that may override any consent needed.

#### EU

*Art. 10* offers “specific considerations for vulnerable populations”, in particular, minors (see Art. 32), incapacitated subjects (see more Art. 31, 28, 29), pregnant or breastfeeding women (see Art. 33), the participation of specific groups or subgroups of subjects, where appropriate, specific consideration shall be given to the assessment of the application for authorization of that clinical trial on the basis of expertise in the population represented by the subjects concerned. Art. 34 also covers national measures for participants performing mandatory military service, persons deprived of liberty, persons who, due to a judicial decision, cannot take part in clinical trials, or persons in residential care institutions.

### Favorable risk-benefit ratio

#### Egypt

Clear specifications of the Principal investigator requires all the consideration about risk-benefit ratio (both at the physical and psychological level), ensuring dignity and health, adding a note for specific attention to reducing side effects (Art. 18; 6). Another layer of risk reduction is the provision to evaluate preclinical medical research (Ch. 5 Art. 10), the provision of health insurance coverage of any research participant (Ch. 7 Art. 18 §9), and ensuring that the research organization will be able to attend properly to research participants’ health needs in case adverse effects or health risks ensuing from the clinical trial (Ch. 11).

#### Sweden

According to the Swedish legislation, the necessary condition for approving research is that fundamental personal freedoms, and human rights are respected. While Section 9 states that research may be approved if its scientific value outweighs the risks to research participants, Section 8 specifies that their welfare must be prioritized over the needs of society and science. Section 10 states that research should be conducted only if its expected result cannot be achieved in another way that involves less risk to the health, safety, and personal integrity of research participants.

#### France

Favorable benefit-risk ratio was refined with the inclusion of “new facts” issues that appeared with Loi Jardé. The most important point to emphasize is that the sponsor will be responsible for the care and necessary costs ensued from severe side effects, if they occur. These include both biomedical research (R1) as well as interventional minimal risk research (R2).

#### EU

Art. 6 ensures that risks (minimization, safety measures) and inconveniences for the subject are considered and reduced for medicinal products and interventions compared to normal clinical practice. Suspected unexpected serious adverse reactions and annual reporting are strictly regulated at the EU level. Under the CTR, the committees are informed regarding suspected unexpected serious adverse reactions that are reported pursuant to the CTR as well as the annual report submitted to the European Medical Agency (Art. 44.3).

### Independent review

#### Egypt

Independent review will be implemented by a REC (Art. 1, 24), and it will protect the rights of participants, review the research protocol, decide on approval, amendments or renewal of the research, and lastly monitor the research (All this is in accordance to the executive regulations of law art 8). The specifics of the review process are detailed in several articles (Ch. 2 Art. 4: REC; Ch. 3 Art 1 to 4).

#### Sweden

According to Section 6, independent review is mandatory whenever the research involves a physical intervention or involves affecting or risk harming the research participant physically or psychologically. Independent review is also required in the case of studies involving biological material taken from a living person and can be traced to that person. The same article emphasizes the principal investigator’s responsibility, who must take measures to prevent research from being carried out in violation of the law. Section 25 sets out the organization of the authority providing an independent review, i.e., the Ethics Review Authority. This is divided into operational regions, each composed of one or more departments according to their areas of expertise. Departments consist of a chairman, who is or has been an ordinary judge, and fifteen other members, of whom ten have scientific competence and five represent public interests, including at least one member who represents one or more patient organizations. The government appoints the chairman and its deputy, while the Ethics Review Authority chooses the other members and their deputies.

#### France

The independent review component is well established with the composition of the Committee for the Protection of Persons and the presence of 39 RECs across the 7 inter-regions committees. The repartition of REC into 2 colleges, one more scientific and the second more patient-related, support independent review, but the designation and recruitment of different REC members (depending on the national or local level, for example) is not clear. For the local levels (Art L1123-1), the text state that the Health Minister CPP for a fixed or undetermined duration and according to the needs. Their members are appointed by the Director General of the regional health agency in which the committee has its headquarters. The committees are completely independent in the performance of their duties. They have legal entity under public law. Committee resources are provided by the State. However, ethical approval can be obtained via institutional committees (in house at some hospitals and universities). Member of the National commission for research involving human need to declare their conflicts of interest (Art. L1123-1-1) which is not clarified for CPP (promotors of the same institution are – per definition- applying to their “in house” ethical committee).

#### EU

Art. 4 refers to the need for prior authorization in accordance with the law of the Member State concerned^6^. The review by the ethics committee may encompass aspects addressed in Part I of the assessment report for the authorization of a clinical trial as referred to in Article 6 and in Part II of that assessment report as referred to in Article 7 as appropriate for each Member State concerned. Article 9 should ensure that the persons validating and assessing the application do not have conflicts of interest, are independent of the sponsor, of the clinical trial site and the investigators involved and of persons financing the clinical trial, as well as free of any other undue influence. A special mention explains that at least one layperson shall participate in the assessment.

### Informed consent (IC)

#### Egypt

Among the very first articles (Art. 1; 21), Egypt’s law provides a definition of IC, promoting its engagement into this ethical procedure “the written expression based on complete voluntary freewill of the person with full legal capacity, and it includes his explicit consent as a signature and a fingerprint to participate in clinical medical research, after all aspects of the research are explained to him, and in particular the potential effects or harms that may impact his/her decision to participate[…]”. The exception of obtaining IC is detailed in executive regulations (Ch. 5, Art. 12; 3). More specific consideration is also represented in other sections of the law: in Ch. 7 Art. 17 § 2: obtaining IC is mandatory. In Ch. 10, Art. 23 §2, IC is required for data usage and for further research. Furthermore it provides specification for consent of data usage.

#### Sweden

Section 17 states that, in line of principle, research can only be performed if the research participant has voluntarily and explicitly consented in a documented way after receiving adequate and specific information. Section 16 describes what the fundamental pieces of information are. In cases where a research participant is in a dependent relationship with components of the research team or if the research participant has difficulties asserting their right, Section 14 states that issues of information and consent must be given special attention. Specific recommendations are provided in the case of minors or if the research participants turned 15 years (Section. 18). Sections 20, 21, and 22 list under what circumstances research can be performed without consent (illness, mental disorder, a weakened state of health, or any other similar condition of the research participant prevents their consent from being obtained).

#### France

IC regulations state that “Consent is free, informed and (voluntary) emphasizing the importance of individuals providing explicit agreement in various legal situations. It must be written for category 1 studies and may be oral for category 2 studies (but must be recorded in the medical file). For category 3 research and for studies on data collected in the course of normal care, the rule is that the patient “must not object.” No research mentioned in 1° of Art. L. 1121-1 may be carried out on a person without his or her free IC, given in writing after the person has been provided with the relevant information. Where it is impossible for the person concerned to express his or her consent in writing, it may be attested by the trusted support person provided for in Art. L. 1111-6, by a family member, or, failing this, by one of the person's close relations, provided that this trusted person, family member or close relation is independent of the investigator and the sponsor. Specific recommendations are provided for minors (under 18 solely). Article 4 is also providing details about the case where the participant cannot express consent and is not under guardianship. There are also options for “collective consent,” but they are only available for interventional research with minimal risk (epidemiologic search).

#### EU

Art. 7 mentions the need for compliance with the requirements for IC as set out in Art. 29, explicating the regulations about written IC. A specific regulation has also been dedicated in Art. 30 for cluster trials. This specification states that “Where a clinical trial is to be conducted exclusively in one Member State, that Member State may, without prejudice to Art. 35, and by way of derogation from points (b), (c), and (g) of Art. 28(1), Art. 29(1), point (c) of Art. 29(2), 29(3), (4) and (5), points (a), (b) and (c) of Art. 31(1) and points (a), (b) and (c) of Art. 32(1), allow the investigator to obtain IC by the simplified means set out in paragraph 2 of this Article, provided that all of the conditions set out in paragraph 3 of this Article are fulfilled.”

### Respect for participants

#### Egypt

Egyptian law provides protection of privacy and data (Art. 12; 2), adequate information of researcher participants (Art,.15; 2. Ch. 5; Art 18:5), protection from publicity (Art. 15;3) together with a straightforward explanation of requirements to respect withdrawal of consent (Art. 2, 1) and compensation aspects (Art. 20:9, 10). The details of non-induced participation (for money or reward) could also be understood as a measure of respect for recruited participants (Art. 14).

#### Sweden

Section 1 states that its purpose is to protect the individual and respect human dignity in research. This is reaffirmed under Section 7. Noteworthy, according to Section 40, some exceptions can be made with regards to requiring consent or processing of data if this is requested by the government or another authority. This is only possible if it is clear that the research does not entail any appreciable risk to an individual's health or safety or pose an infringement on an individual's integrity.

#### France

The respect for study participants was unclear in the text and focuses more on fair selection and risk protection of research participants. One specific element regarding the participant protection of “a deceased person, in a state of brain death, without his or her consent expressed during his or her lifetime or through the testimony of his or her family” (Art. L1125-13).

#### EU

Article 28 prescribes general rules that must be met for a clinical trial to be lawfully conducted. This article clarifies that benefit to the participants, IC, right to mental and physical integrity, minimal pain or risk, guaranteed medical care, and no undue influence (including financial) are the basis for any medical research. EU regulation Article 28 of Regulation (EU) No 536/2014 of the European Parliament and of the Council, Article 2 make sure that withdrawal is free of constraints and will not have repercussions nor affect the participants' rights and care, but also makes sure that the withdrawal of consent does not affect the data collected prior to withdrawal.

## Discussion

Our results show that the Egyptian law fulfills the ethical requirements for human subject research and is comparable to the French, Swedish and EU regulations.

Detailing the results, we also observed that all regulations tended to have a very vague approach to “social values and scientific values” (principle 1). In terms of the fair selection of participants, the Swedish text was probably the vaguest (principle 3), but in general, this principle appeared to be well integrated. All other principles also were well represented in Egyptian law as in the French, Swedish, and EU’s laws (principles 4, 5, 6, 7). We explore in two separate points the results :1) Value and validity (in which principles 1, 2 and 5, with 5 as the way/procedure to reach value and validity), 2) Participant’s protection (principles 3, 4, 6, and 7).

### Values and scientific validity

#### Principle 1, Social values and scientific value

Egyptian regulation suffers from the same issues in clarifying the social value aspect as French, Swedish, and EU regulation. The results from the assessment of social values reveal that most of the regulations struggle to clearly define under what specifications research should serve a social purpose. Social value is often envisioned at the level of cost-effectiveness measurements, and its definition may be difficult to normalize across states and cultures as it refers to “the general concept and practice of measuring social impacts, outcomes, and outputs through the lens of cost” [37]. In Emanuel et al’s vision, it is composed of the a) ensured benefit b) value for the prospective beneficiaries, c) dissemination of the results via long-term collaborative strategy d) avoiding to undermine the community’s existing healthcare [38]. Furthermore, these results question the importance of social value in research per se, for example, in specific areas where the social value aspect cannot be considered as an overarching guide. Recent debates questioned, for example, the justice and egalitarian arguments that can arise from questioning the social value of research [39, 40] depending on how innovative and impactful on a societal level the research is.

#### Principle 2, Scientific validity

Egyptian law, French, Swedish, and EU regulation, tries to specify the scientific validity mostly via their REC members selection. The general implementation of a control mechanism and ethical review board’s competences question the real level of expertise or education that these members do have in order to review research protocols or scientific methods. For example, there is not a clear consensus about the need for specific competencies in order to have balanced and non-biased decisions in ethical vetting because [41] as different ethical reviewers will raise different concerns. In their results, they confirmed that the main influencing factor in readers-queries was the profession, with scientific validity issues being more frequently asked by scientific reviewers, whereas ethical issues were more frequently pointed out by ethicists.

Depending on the system and general community functioning, the selection of research ethics committees members can put into question the non-biased nature of assessment of scientific validity. For instance, in France, the members of the Nation Ethical Committee, who are responsible for offering direction to all REC, are "selected" or "designated" by the President of France. The same question could also be raised in Egypt were Central intelligence members are sitting at the National REC (the Supreme Council). The impact of politics and social politics in presumably non biased procedure is also rising with the use of preference studies to inform policy making and including patient’s advocacy in decisions boards as it can have a role in decision makings [42] providing advances in shared decision making but also leveraging non-biased decisions making as there is still not a unified definition of such processes [43] and for example, research shows that methodological standards are often downgraded to provide access to the co-researchers [44].

As it is sometimes defined in science in general, validity should be assessed in an adequate manner across medical field. Scientists refer to scientific quality measures (like in systematic literature review assessements scales for scientific quality) but validity interpretation can be difficult to apprenhend for an heterogeneous group of experts (like an REC). In its very classical definition “The validity of a research study refers to how well the results among the study participants represent true findings among similar individuals outside the study. This concept of validity applies to all types of clinical studies, including those about prevalence, associations, interventions, and diagnosis”, scientific validity could look easy to apprehend but even then, just having the precision “The validity of a research study includes two domains: internal and external validity” explain the layer of complexity that may not be represented in Emanuel’s principles definition [45]. Going further into the validity explanation and use in ethical consideration in biomedical research should be warranted. For example, some research look at different levels of validity to clarify what one considers as validity (scientific validity may be too vague to refer to permit clear assessment): congruence validity, criterion validities, etc [46]). Wages et al. in 2021 [47], showed the potential for using operating characteristics to inform design’s safety and accuracy in phase I clinical trials that could open the debate around a better definition of scientific validity checks in biomedical research. One part of the issue comes from scientific communities but the scientific validity should be also a matter of concerns for all REC members. The competencies of any participants should be addressed in the REC reviewer’s selection as some questions also arise from the medical field where shared decision-making has been implemented before and some debate remains about the representativeness of patients that do get involved in the medical decision-making [48, 49].

#### Principle 5, Independent review

All ethical regulations, including Egypt’s, ensure that the review system is independent and thus RECs have the power to authorize, follow up and end any research to protect participants [29]. One of the major concerns still not addressed in the regulations is the difficulty to guarantee REC effectiveness in regards to some deficiencies in REC theory and structure [50]. The opportunities to enhance EC efficiency and effectiveness could also depend more on the researcher and the scientific community as Hickey et al., 2022 suggested [51]. Clarifying the collaborative approach across ethics committees and research can be the path to increased medical research efficiency.

### Participant’s protection

#### Principle 3, Fair selection

In the Egyptian regulation, the fair selection of participants is pursued, which can be considered a positive development with respect to the previous regulation proposal, where protection of the rights and welfare of vulnerable subjects were not adequately considered [52]. Generally, one can wonder about the impact of fairness when recruiting for clinical trials. Ongoing discussions emphasize that the fair selection of participants could be a very ethically challenging issue as it is a ground for dilemmas [53], including the levels of “(1) fair inclusion; (2) fair burden sharing; (3) fair opportunity; and (4) fair distribution of third-party risks”. The equal opportunity issue also arose for example in 2022 when French law integrated EU requirements and shifted toward allowing research participants with no access to social security to be part of research [54], offering extended opportunities for participation but putting the question of fairness of selection into question.

#### Principle 4, Favorable risk-benefit ratio

Egyptian regulation, like the four other comparatives, consider “Risk-benefit” as a sine qua none principle and the clarity of Helsinki’s declaration [55]. However, no regulation mentioned the potential for under-reporting harms depending on what one considers to be “harm”. One of the aspects that are often under-considered is psychological harm, as even clinical trials in the field tend not to report psychological harm (compared to physical adverse effects of drugs in clinical trials) as can be noted in research by [56, 57].

#### Principle 6, Informed consent

Informed consent is implemented overall, and as for principle 5, Egypt complies, like all comparatives to this standard practice. Even if a variety of informed consent exists, the law sticks to written consent without specifying the potential for renewed consent, broad consent, or other approaches [58] and the “blanket consent” potential.

#### Principle 7, Respect for recruited participants and study communities

Respect for participants reflects respecting autonomy across health care and research systems that appear to be consistent across the 4 regulations. Egyptian law places an equivalent emphasis on this principle as French law and is comparable to Swedish and EU regulation. All these regulatory frameworks effectively incorporate this principle into their bioethical laws. Heightened attention still needs to be paid to respect at different levels, such as for gender issues [59], or/and ethnicity [60, 61].

### Limitations

Our study contains limitations. The first one is that we looked for expressis verbis statements that limit the apprehension of the full corpus of laws application in a specific context. Indeed, some implicit references could counterbalance our conclusions. For instance, the CTR underscores the overarching objective of promoting the social value by enhancing health, even if not explicitly stated. Nevertheless, at the clinical level, practitioners may not have comprehensive access to all regulations and are likely to rely on referenced texts in the ethical application specific to their country. The lack of clarity (or complex implicit references) may hinder comprehension and result in a complex implementation process. Utilizing the principles proposed by Emanuel et al. may also be presented as a limitation as this analytical foundation may not comprehensively encapsulate the nuances inherent in the examined legal frameworks. Another limitation could be posited in the fact that the analysis may not fully reflect the influence of cultural and social variations among the three countries. Further research would need to also assess the overall structure of ethical procedure in each country and their organization (from Supreme councils, regional entities, national unified procedures, etc).

## Conclusion

In conclusion, the Egyptian law in comparison to French, Swedish, and the connected EU regulations reveals its alignment with Emanuel et al.’s principles. However, several common challenges and areas of improvement can be sought with regards to each of the ethical principles and thus open the way for further research. The main topic identified via our analysis is the need to clarify and standardize the concept of social value of research, which often focuses on cost-effectiveness measurements and implicitly -not always directly- refers to a very difficult concept to apply [62]. Our second main discussion point highlights concern about the expertise and unbiased decision-making of ethical review boards. Further research is warranted to explore in more detail’s other principles. Overall, these findings highlight the need for continuous improvement and refinement of ethical regulations to ensure the protection of participants and the integrity of research in Egypt and other jurisdictions.

Based on the discussion, the following recommendations can be made for improving research ethics regulations in the countries in our analysis:

1. Clarify and Standardize Social Value: Develop clear guidelines and standards to define and measure the social value of research across different states and cultures. This should include a detailed framework for assessing research's contribution to societal benefits, cost-effectiveness, and its alignment with the long-term healthcare goals of the community.
2. Enhance Scientific Validity: Strengthen the criteria for the selection of Research Ethics Committee (REC) members to ensure they possess the necessary expertise and education to review research protocols and scientific methods effectively. This includes establishing more rigorous competency requirements and providing ongoing training to ensure balanced and non-biased decision-making in ethical approvals.
3. Improve Participant Protection: Emphasize the fair selection of participants by addressing ethical challenges and ensuring equitable opportunities for participation. This involves revising existing regulations to better protect the rights and welfare of vulnerable subjects and to promote fairness in participant selection.
4. Increase REC Effectiveness: Address deficiencies in REC theory and structure to enhance the effectiveness of research ethics committees. This could involve adopting more collaborative approaches between review boards and researchers, and ensuring that ethics committees have the authority, independence, competences and resources needed to oversee research effectively.
5. Promote Respect for Participants: Ensure that all research activities respect the autonomy and dignity of participants. This entails paying heightened attention to issues of gender, ethnicity, and other factors that may affect participants' experiences in research settings.
6. Further Research: Encourage further research into the nuances of ethical principles beyond those identified by Emanuel et al., to better understand the cultural and social variations that may affect the implementation of ethical guidelines in different jurisdictions and cultural contexts.

### Supplementary Information

**Additional file 1: Appendix 1.** Comparative table with references to law texts.

## Data Availability

All data generated or analyzed during this study are included in this published article [and its supplementary information files].

## Abbreviations

CS: Code de la santé / Health code
REC: Research Ethics Committee
CTR: Clinical trial regulations
Art.: Article
Ch.: Chapter
§: Paragraph
LMIC: Low to medium income country
EU: Europe
CNIL: Commission nationale de l'informatique et des libertés / national commission for computer science and freedoms
REC: French specific will stand for
CPP: Comité de Protection des personnes / person’s protection committee
IC: Informed consent

## Footnotes

1 US Department of Health and Human Services. (1979). The Belmont Report: Office of the Secretary, Ethical Principles and Guidelines for the Protection of Human Subjects of Research, the National Commission for the Protection of Human Subjects of Biomedical and Behavioral Research.

2 « World Medical Association Declaration of Helsinki: Ethical Principles for Medical Research Involving Human Subjects », *JAMA*, vol. 310, n^o^ 20,‎ 27 novembre 2013, p. 2191–2194

3 Department of Health. (2015). Ethics in health research: Principles, processes and structures.

4 The composition of the ethics committees remains to be decided by a member state.

5 In France, the REC acronym coul refer more to the High National Ethical Committee, which issues recommendations on a societal scale. It is important to note that this committee is distinct from the day-to-day oversight of research ethical applications or clinical trials. The latter responsibility primarily falls within the purview of internal ethical committees situated within hospitals or universities, which have the authority to grant approvals. Externally, the (Comité de Protection des Personnes or CPP) serves as the most pertinent entity, akin to a Research Ethical Committee (REC); hence, we have opted to utilize the acronym REC for clarity.

6 The effect of the decision is, nonetheless, strong. Where an ethics committee has issued a negative opinion, on the condition that rules that are valid for the entire Member State apply, that Member State has a duty to refuse to authorise a clinical trial (Article 8.4, for extended authorisations see Article 14.10, for substantial modifications for the assessment report, see Articles 19, 20 and 23).
