# Supplementary material for: A comparative ethical analysis of the Egyptian clinical research law
Source: BMC Med Ethics. 2024 Apr 30;25:48. doi: 10.1186/s12910-024-01040-0 (PMC11059645; doi:10.1186/s12910-024-01040-0)
Supplement: Supplementary file 1 — Additional file 1: Appendix 1. Comparative table with references to law texts. [file 12910_2024_1040_MOESM1_ESM.docx]

Appendix 1: Comparative table with references to law texts

| **Emanuel et al.,, 2000** | **French Law** | **EU Regulation** | **Egypt the law and the executive regulations published 2022** | **Sweden** |
| --- | --- | --- | --- | --- |
| **Social and scientific value**  The law states anything on evaluation of a treatment, intervention, or theory that will improve health and well-being or increase knowledge.  (- Specify the beneficiaries of the research—**who.**  -  Assess the importance of the health problems being investigated and the prospective value of the research for each of the beneficiaries—**what.**  - Enhance the value of the research for each of the beneficiaries through dissemination of knowledge, product development, long-term research collaboration, and/or health system improvements.  -  Prevent supplanting the extant health system infrastructure and services.) | When the European regulation comes into force (initially planned for 2016, but in practice when the single European portal is made available), there will be a contradiction between the latter and the Jardé law, particularly concerning the typology of research. For the time being, it is accepted that the European regulation will apply to drug trials, while for other fields of research the Jardé law should apply. This is under the Code de la Santé.  **Article L1121-2 Modifié par LOI n°2012-300 du 5 mars 2012 - art. 1 (V)**No research involving the human person may be carried out :- if it is not based on the latest scientific knowledge and on sufficient pre-clinical experimentation ;[...]  - if the research does not aim to extend scientific knowledge of the human being and the means likely to improve his condition;  - if the research involving the human being has not been designed in such a way as to minimize pain, discomfort, fear and any other foreseeable inconvenience related to the disease or the research, considering in particular the degree of maturity of minors and the capacity for understanding of adults who are not in a position to give their consent.”  **Article L1121-16** from Code de Santé offer public access to results aside from National Defense matters. | **Article 3** **General principle:** A clinical trial may be conducted only if: (a) the rights, safety, dignity and well-being of subjects are protected and prevail over all other interests; and (b) it is designed to generate reliable and robust data.  **Article 6:** b) i) the relevance of the clinical trial, including whether the groups of subjects participating in the clinical trial represent the population to be treated, or if not, the explanation and justification provided in accordance with po **Article 3a** is not more about the principle of Respect for potential and enrolled subjects. Similarly, 3b seems to me more about Scientific validity than Social and scientific value.  Moreover, it may be noted that in order to ensure compliance with the EU regulation, member state and Union inspections are contemplated (taking compliance with the EU regulation as a token of good research for society and for science). **See art.78 and 79.** | With the exception of medical interventions for local diseases that do not exist in the country of origin of the medical intervention and rare diseases, medical research on any of them is allowed in the Arab Republic of Egypt beginning from phase 2, and as approved by the Supreme Council” **Art 10**  -” Commitment to publish the results of the medical research in one of the specialized scientific journals after its completion” PI resp **ch 5, art 18.**  “quality, and ensuring that medical research results are mined, documented and published,” sponsor resp, **ch  8 art 20.**  - no  - Setting standards, controls/rules and regulations for ethics of medical research to protect humans, their samples and their data, and reviewing them as  necessitated by the national interest and international scientific developments **Art 7; 2 - Ch 2 Art 10** in executive regulations - SC will be in charge of establishing standards, regulations and guidelines for ethics of clinical trials and review them periodically as seen fit for national interests and the developments in the international scientific field.  **Executive regulations art 24-** dissemination of results of research in scientific articles. | No provisions on social value. |
| **Scientific validity**  Use of accepted scientific principles and methods, including statistical techniques, to produce reliable and valid data  (- Ensure that the scientific design of the research realizes social value for the primary beneficiaries of the research.  -  Ensure that the scientific design realizes the scientific objectives while guaranteeing research participants the health-care interventions to which they are entitled.  - Ensure that the research study is feasible within the social, political, and cultural context or with sustainable improvements in the local health-care and physical infrastructure.) | The stakeholders of Biomedical Research according to French system are guaranteeing the methods, analysis and reliability of the research and under the surveillance of qualified personal **Art. L 1121-3** and after the approval has been given by an ethic committee names CPP. The scientific guaranties are the responsibility of several actors in research project, namely:  **Promoter:** a natural or legal person (pharmaceutical industry, university hospital, etc.) who takes the initiative for biomedical research. It ensures the implementation, management and verification of financing at different level (listed here).  **Investigator:** a natural person who directs and supervises the performance of the research in an authorized location. This person must be a registered physician. If there are several investigators, a coordinator may be designated by the sponsor.  **CPP**: Comité de Protection des Personnes (Personal Protection Committee). There is one or more committees per region. It is an independent body, composed in such a way as to guarantee its independence and the diversity of its skills in the biomedical field and with regard to ethical, social, psychological and legal issues. It has legal personality and includes representatives of associations of patients or users of the health system approved and designated under the provisions of article L. 1114-1 of the public health code. The CPP comprises fourteen full members appointed by the representative of the state in the region in which the committee has its headquarters. The favorable opinion of a CPP is mandatory to be able to start a research.  **CNIL:** Commission Nationale Informatique et Liberté. Body ruling on the processing of personal data for the purpose of research in the field of health (July 1994 law).  **CEREES:** Comité d'Expertise pour les Recherches, les Etudes et les Evaluations dans le domaine de la Santé. It issues an opinion on the methodology, on the need to use personal data, on the relevance of these data in relation to the purpose of the processing and, if necessary, on the scientific quality of the project.  **Competent authority (ANSM in France)**: It ensures the quality and safety of the products used as well as the safety of the persons who take part in the research.  **Article L.1121-8-1:** The person affiliated to the social security system or that benefits from such system may be sollicited for research. **Arti. L1123-7** CPP has regulated obligation to control for scientific validity. | **Article 4**  **Prior authorisation**  A clinical trial shall be subject to scientific and ethical review and shall be authorised in accordance with this Regulation  **Article 6:**  b)i) the reliability and robustness of the data generated in the clinical trial, taking account of statistical approaches, design of the clinical trial and methodology, including sample size and randomisation, comparator and endpoints; | -“in accordance with internationally recognized ethical standards for research.” def of clinical research**, article 1, #2**  “and apply ethical principles that must be followed in this regard” def of IRB, **art 1, #24**  Setting standards, controls/rules and regulations for ethics of medical research to protect humans, their samples and their data, and reviewing them as necessitated by the national interest and international scientific developments **Art 7; 2**  Ch 2 Art 10 in executive regulations - SC will be in charge of establishing standards, regulations and guidelines for ethics of clinical trials and review them periodically as seen fit for national interests and the developments in the international scientific field.  **Executive regulations:** ensuring the PI and co PI have the necessary qualifications, medical, ethical and research expertise to carry out their proposed research**. Ch 5 art 22.**  **Chap 3 art  6** provided a detailed list of the competences required **§2**  **Chap 4 art 9.** Scientific review | **11 §** Research may only be approved if it is to be carried out by or under the supervision of a researcher who has the necessary scientific competence.  **Verksamhetsregioner och avdelningar av Etikprövning 25§**  En avdelning ska bestå av en ordförande och femton övriga ledamöter. Av de övriga ledamöterna ska tio ha vetenskaplig kompetens och fem företräda allmänna intressen.  Ethics Review Authority: A department shall consist of a chairman and fifteen other members. Of the other members, ten must have scientific competence and five must represent public interests  **Beslutsförhet** **26 §** En avdelning inom Etikprövningsmyndigheten är beslutsför, när ordföranden och minst åtta övriga ledamöter är närvarande. Av de övriga ledamöterna ska minst fem ha vetenskaplig kompetens och minst två företräda allmänna intressen. Ledamöter med vetenskaplig kompetens ska alltid vara i flertal, när ett ärende avgörs.  Of the other members, at least five must have scientific competence and at least two must represent public interests. Members with scientific competence must always be in the majority when a case is decide. |
| **Fair selection of study population**  Selection of subjects so that stigmatized and vulnerable individuals are not targeted for risky research and the rich and socially powerful not favored for potentially beneficial research  (- Select the study population to ensure scientific validity of the research.  -  Select the study population to minimize the risks of the research and enhance other principles, especially collaborative partnership and social value.  - Identify and protect vulnerable populations) | Since 1988: Huriet-Serusclat Law (French law) defined the responsibility of a representative to take responsibility for the research. It’s the first time the law defines a “Sponsor”: natural or legal person who takes the initiative of a biomedical research. At the same time, this law created the clear Obligation to submit clinical research protocols to an advisory committee for the protection of individuals. It is also stated that “Free and informed consent of the patients involved in clinical research” and penalties will apply in case of non-compliance.  Specific consent rules for research involving vulnerable persons In order to overcome this dilemma, the legislator first of all the principle of prohibiting the solicitation of such persons to participate to participate in biomedical research when research of comparable effectiveness can be carried out be carried out on another category of the population" **(Articles L. 1121-5 to (Articles L. 1121-5 to L. 1121-8 of the Code de la santé Code - CSP)**. Their participation in a research project must therefore constitute the exception. It then carefully defined, for each category of vulnerable category of vulnerable persons, the situations and conditions under conditions under which they may be included in a biomedical in a biomedical research protocol. Finally, it has provided for specific information and consent procedures for each situation. Thus, with regard to minors, **article L. 1122-2 of the** **Public Health Code** provides that, if the authorization is given by the holders of parental authority, minors receive information parental authority, minors receive information adapted to their capacity of understanding. They are consulted to the extent that their condition permits and their personal consent to participate in biomedical research is sought. With regard to persons who are not in a condition to consent the legislator has distinguished several situations – protected adults, adults who are unable to express their consent and consent and who are not subject to a legal protection measure of legal protection **(article L. 1122-2 of the CSP)** and  persons unable to express their consent and who are in an emergency situation **(article L. 1122-1-2 of the CSP**) - while applying specific consent regimes to them. It is therefore essential not to confuse these situations. Despite the precautions taken by the legislator to protect these  categories of persons, the implementation of these the practical application of these provisions raises particular difficulties depending on the type of population, the situation in which they situation in which they find themselves and the type of research in which they are participating. A typical example in this sense is research in emergency situations. Involving people who are, for the most part, in no condition to give their consent.  **Exceptional circumstances for patient information and consent collection** They are limited and subject to heavy legal requirements. For adults who are protected or unable to express their consent (coma, [senile dementia](https://www.sciencedirect.com/topics/medicine-and-dentistry/senile-dementia), psychiatric reasons, or enfeebled patient): an authorisation is required from the tutor or the curator for protected adults and minors **(art. L. 1122-2 II, §3**), or from the designated person of trust, a family member by default, or a person with strong and reliable ties to the patient (as a last resort) (**art. L.1122-2 PHC**). People deprived of their freedom by legal or administrative decisions or people benefiting from psychiatric care unable to express their consent can only participate to categories 1 or 2 researches if the direct expected advantage for these people justifies the foreseeable risks, or if there is expected advantage for people in the same situation and the study cannot be carried out on other types of subjects. In this last case, foreseeable risks and constraints must be minimal (**Art. L. 1121-6 PHC**). **Foreigners**; in the preliminary part it is mandatory to mention that studies covered by Jardé law and carried out on human subjects who do not possess a social security number are forbidden, except for category 3 researches. Thus, observational researches on migrant populations aiming to facilitate the screening of rare or emergent diseases on national soil are possible for instance. By way of exception, the law allows this population to participate in category 1 and 2 researches (**art. L.1121-8-1 PHC**) and therefore benefit from innovative care protocols whatever their situation, insofar as the expected advantage is considered a justification of foreseeable risks. The risk of operating on a specific type of population is submitted to the control of CPPs who are meant to authorise these researches. **Urgency** The law provides an exemption from the obligation to obtain the person's consent by collecting the consent of a family member or the designated person of trust if those are present. If none are present, the patient, the family or the person of trust are informed as soon as possible of the carrying out of the research and the consent is then required for the continuation of the study (**art. L.1122-1-2 PHC**). In case of vital emergency, the protocol can allow a release from this obligation. Only the CPP can authorise such derogation. In this context, the patient must be informed and their consent must be obtained as soon as they regain consciousness and enough capacity for discernment. They must consent to the continuation of the research on themselves and be informed of their right to object (delayed consent). Conviction is possible for not attempting to seek consent from an emergency hospitalized patient's family member when the family is present and the protocol requires it. **Collective consent collection** For some interventional researches entailing minimal risks (category 2), it is possible to deliver the information collectively and not seek to obtain consent when research methodological expectations are incompatible with collection of express consent (art. **L.1122-1-3 PHC**). The request to be released from the obligation to inform every subject individually must be approved by the CPP. Example: [epidemiological research](https://www.sciencedirect.com/topics/medicine-and-dentistry/cohort-effect). **Research on a person's biological characteristics** Examination of a person's genetic characteristics can only be carried out for medical or scientific research purposes. Express consent must be obtained from the person in writing before carrying out the examination and after informing them of its nature and purpose. Consent must reflect the purpose of the examination and is freely revocable at any stage (art. 16-10 Civil Code). By derogation to article 16-10 of the Civil Code, the law provides that the examination of a person's genetic characteristics for scientific research can be carried out with elements from the person's body extracted for other purposes when the person has been duly informed of the project and did not object (**art L.1131-1-1 PHC**). Therefore, the protocol consist in informing the person at the time of the samples collection that research on their genetic characteristics could be carried out and take note of their non-objection. It is possible to be released from the information obligation when the person cannot be found (**art. L1131-1-1 PHC**). The promoter must however consult with a CPP before starting the study in order to confirm that the person did not object to the examination of their genetic characteristics and obtain an opinion on the scientific interest of the study at hand. **Research on minors and matters of parental consent** In principle, both parents must consent to any interventional research on their child, whether they entail minimal risk or not (categories 1 or 2) (**art. L.1122-2 II PHC**).There are two exceptions:-the present parent can give their consent only when research entails minimal risks (category 2) but;-the minor must not qualify as a healthy volunteer,-the collection of the other parent's consent is incompatible in terms of time frame with the methodological requirements of the study with regards to its objectives (**art. L. 1122-2 II PHC**);- no authorisation shall be sought for observational researches carried out on a minor (category 3). However, informed parents can object to it. Law extends here the criminal sanctions applicable to promoters who carry out clinical trials without previously obtaining the mandatory authorisations for all interventional and observational researches (**art. L.223-8 Penal Code**) | **Article 10** **Specific considerations for vulnerable populations 1.** Where the subjects are minors, specific consideration shall be given to the assessment of the application for authorisation of a clinical trial on the basis of paediatric expertise or after taking advice on clinical, ethical and psychosocial problems in the field of pediatrics **2.** Where the subjects are incapacitated subjects, specific consideration shall be given to the assessment of the application for authorisation of a clinical trial on the basis of expertise in the relevant disease and the patient population concerned or after taking advice on clinical, ethical and psychosocial questions in the field of the relevant disease and the patient population concerned. **3.** Where the subjects are pregnant or breastfeeding women, specific consideration shall be given to the assessment of the application for authorisation of a clinical trial on the basis of expertise in the relevant condition and the population represented by the subject concerned **4.** If according to the protocol a clinical trial provides for the partici­pation of specific groups or subgroups of subjects, where appropriate, specific consideration shall be given to the assessment of the application for authorisation of that clinical trial on the basis of expertise in the population represented by the subjects concerned. **5.** In any application for authorisation of a clinical trial referred to in **Article 35,** specific consideration shall be given to the circumstances of the conduct of the clinical trial.  **Article 31 Clinical trials on incapacitated subjects: 1.** In the case of incapacitated subjects who have not given, or have not refused to give, informed consent before the onset of their incapacity, a clinical trial may be conducted only where, in addition to the conditions set out in **Article 28,** all of the following conditions are met:(a)  the informed consent of their legally designated representative has been obtained; (b)  the incapacitated subjects have received the information referred to in **Article 29(2)** in a way that is adequate in view of their capacity to understand it; (c)  the explicit wish of an incapacitated subject who is capable of forming an opinion and assessing the information referred to in **Article 29(2)** to refuse participation in, or to withdraw from, the clinical trial at any time, is respected by the investigator; (d)  no incentives or financial inducements are given to the subjects or their legally designated representatives, except for compensation for expenses and loss of earnings directly related to the participation in the clinical trial;(e)  the clinical trial is essential with respect to incapacitated subjects and data of comparable validity cannot be obtained in clinical trials on persons able to give informed consent, or by other research methods; (f)  the clinical trial relates directly to a medical condition from which the subject suffers;(g)  there are scientific grounds for expecting that participation in the clinical trial will produce: (i)  a direct benefit to the incapacitated subject outweighing the risks and burdens involved; or (ii)  some benefit for the population represented by the incapacitated subject concerned when the clinical trial relates directly to the life-threatening or debilitating medical condition from which the subject suffers and such trial will pose only minimal risk to, and will impose minimal burden on, the incapacitated subject concerned in comparison with the standard treatment of the incapacitated subject's condition. 2. Point (g) (ii) of paragraph 1 shall be without prejudice to stringent national rules prohibiting the conduct of those clinical trials on incapacitated subjects, where there are no scientific grounds to expect that participation in the clinical trial will produce a direct benefit to the subject outweighing the risks and burdens involved. **3**. The subject shall as far as possible take part in the informed consent procedure. **Article 32 Clinical trials on minors** 1. A clinical trial on minors may be conducted only where, in addition to the conditions set out in **Article 28**, all of the following conditions are met: (a)  the informed consent of their legally designated representative has been obtained; (b)  the minors have received the information referred to in **Article 29(2)** in a way adapted to their age and mental maturity and from investigators or members of the investigating team who are trained or experienced in working with children; (c)  the explicit wish of a minor who is capable of forming an opinion and assessing the information referred to in **Article 29(2**) to refuse participation in, or to withdraw from, the clinical trial at any time, is respected by the investigator (d)  no incentives or financial inducements are given to the subject or his or her legally designated representative except for compensation for expenses and loss of earnings directly related to the participation in the clinical trial; (e)  the clinical trial is intended to investigate treatments for a medical condition that only occurs in minors or the clinical trial is essential with respect to minors to validate data obtained in clinical trials on persons able to give informed consent or by other research methods; (f)  the clinical trial either relates directly to a medical condition from which the minor concerned suffers or is of such a nature that it can only be carried out on minors; (g)  there are scientific grounds for expecting that participation in the clinical trial will produce: (i)  a direct benefit for the minor concerned outweighing the risks and burdens involved; or (ii)  some benefit for the population represented by the minor concerned and such a clinical trial will pose only minimal risk to, and will impose minimal burden on, the minor concerned in comparison with the standard treatment of the minor's condition.  2.The minor shall take part in the informed consent procedure in a way adapted to his or her age and mental maturity. 3. If during a clinical trial the minor reaches the age of legal competence to give informed consent as defined in the law of the Member State concerned, his or her express informed consent shall be obtained before that subject can continue to participate in the clinical trial.  **Article 33 Clinical trials on pregnant or breastfeeding women** A clinical trial on pregnant or breastfeeding women may be conducted only where, in addition to the conditions set out in Article 28, the following conditions are met: (a)  the clinical trial has the potential to produce a direct benefit for the pregnant or breastfeeding woman concerned, or her embryo, fetus or child after birth, outweighing the risks and burdens involved; or (b)  if such clinical trial has no direct benefit for the pregnant or breast­ feeding woman concerned, or her embryo, fetus or child after birth, it can be conducted only if: (i)  a clinical trial of comparable effectiveness cannot be carried out on women who are not pregnant or breastfeeding; (ii)  the clinical trial contributes to the attainment of results capable of benefitting pregnant or breastfeeding women or other women in relation to reproduction or other embryos, fetuses or children; and (iii)  the clinical trial poses a minimal risk to, and imposes a minimal burden on, the pregnant or breastfeeding woman concerned, her embryo, fetus or child after birth; (c)  where research is undertaken on breastfeeding women, particular care is taken to avoid any adverse impact on the health of the child; and (d)  no incentives or financial inducements are given to the subject except for compensation for expenses and loss of earnings directly related to the participation in the clinical trial. **Article 34** Additional national measures . Member States may maintain additional measures regarding persons performing mandatory military service, persons deprived of liberty, persons who, due to a judicial decision, cannot take part in clinical trials, or persons in residential care institutions. | To choose the research subjects with complete impartiality, and to determine their appropriate number according to the approved research plan (protocol). **Art 17; 7 (PI resp)**  -It may not be permitted to conduct (limit) medical research to a specific group of people or vulnerable populations unless the research is necessary and addresses their specific diseases, and providing the scientific and ethical justifications for their enrollment, and provided that informed consent is obtained from each of them. If the medical research is to be conducted on one of the vulnerable populations, the consent of the two parents, or whoever has the legal guardianship in the event of the death of one or both of the parents, or the legal representative must be obtained, in accordance with the rules and procedures specified by the executive regulations of this law. **art 3**  **Chap 5 art 13.14:** research subject may not participate in another medical research  14. prohibited to induce research subject to participate | **14 §** If a research person is in a dependent relationship with the research principal or a researcher, or if the research person can be assumed to have particular difficulties in asserting his right, issues of information and consent must be given special attention during the ethics review. |
| **Favorable risk-benefit ratio**  Minimization of risks; enhancement of potential benefits; risks to the subject are proportionate to the benefits to the subject and society  -  Assess the potential risks and benefits of the research to the study population in the context of its health risks.  -  Assess the risk-benefit ratio by comparing the net risks of the research project with the potential benefits derived from collaborative partnership, social value, and respect for study populations) | The implementation of the Jarde' law also entails changes in vigilance during clinical research. Indeed, in addition to the unexpected serious undesirable events (EIGI) the notion of "new facts" appears (article **L1123-10**). These new facts are defined by: " toute nouvelle donnée” (any new data) that may lead to a re-evaluation of the relationship between the benefits and risks of the research or of the product subject to the research, to modifications in the changes in the use of this product, the conduct of the research, or of the materials related to the research, or to suspend the research, or to suspend or discontinue or modify the research protocol or similar research (**R. 1123-46)** ". In the event of an IGRA or new facts, the investigator and the sponsor must warn without delay the ANSM as well as the CPP and the director of the ARS, without delay. The most important point to emphasize is that vigilance extends to care and therefore to interventional studies with minimal risk and constraints (R2). The authorization of category 1 research is given by the Agence the National Agency for the Safety of Medicines and Health Products (ANSM), which is competent to evaluate the methodology used. The National Agency for the Safety of Medicines and Health Products (ANSM) was created by the law of December 29, 2011 on the reinforcement of the sanitary safety of medicines and health of medicines and health products. It replace the Agence française de sécurité sanitaire du médicament et des produits de santé (Afssaps). The two main missions of the ANSM are to ensure fair access to innovation for all patients and to guarantee the safety of health products from initial trials to post-marketing authorization. The ANSM issues an authorization for category 1 trials with regard to the safety of the persons who take part in the research. In the case of research involving a health product, the ANSM evaluates the product, the ANSM assesses its relevance, while the CPP gives an opinion on the conditions of validity of this research. The ANSM is also competent authority to monitor and inspect category 1 research. This is a health police power, in case of risk for the persons included or likely to be included in the research if their safety is not guaranteed. Category 2 and 3 research is subject to information to the ANSM (sending the summary and the favorable opinion of the of the CPP.)  The Code de la santé was already clear about “- if the foreseeable risk incurred by the persons who take part in the research is out of proportion to the expected benefit for these persons or the interest of this research;[...]The interests of the persons who take part in research involving the human person always take precedence over the sole interests of science and society.([**Article L1121-2**](https://www.legifrance.gouv.fr/codes/article_lc/LEGIARTI000025457486)) | **Article 6**  b)ii) The risks and inconveniences for the subject, taking account of all of the following:  —  the characteristics of and knowledge about the investigational medicinal products and the auxiliary medicinal products;  —  the characteristics of the intervention compared to normal clinical practice;   —  the safety measures, including provisions for risk minimization measures, monitoring, safety reporting, and the safety plan  —  the risk to subject health posed by the medical condition for which the investigational medicinal product is being inves­tigated; | “Taking the necessary measures to protect the research subject’s life, physical and psychological health and dignity, as well as limiting the side[MOU1]  effects of medical research, with measures such as introducing adjustments to the research plan in the event of severe side effects that may threaten the safety of the research subject. In this situation the Principal Investigator shall notify the research sponsor, the IRB, the Egyptian Drug Authority, and the Supreme Council, each in its own jurisdiction, of the occurrence of these effects and measures that he took to protect the research subject, within twenty-four hours at most.” **Art 18; 6 (PI duties)**  **Chap 5 art 10:** clinical medical research must be preceded by pre-clinical medical research  **Chap 7 art 18 §9** : providing necessary medical care to participating research subjects  **Chap 11 :** requirements of research organization | **8 §** Human rights and fundamental freedoms must always be taken into account during the ethical review, while account must be taken of the interest in new knowledge being developed through research. People's *(I interpret it as research subjects)* welfare must be given priority over the needs of society and science.  **9 §** Research may only be approved if the risks it may entail for the health, safety and personal integrity of research subjects are outweighed by its scientific value.  **10 §** Research may not be approved if the expected result can be achieved in another way that involves less risk to the health, safety, and personal integrity of research subjects. |
| **Independent review**  Review of the design of the research trial, its proposed subject population, and risk-benefit ratio by individuals unaffiliated with the research  (- Ensure public accountability through reviews mandated by laws and regulations.    -  Ensure public accountability through transparency and reviews by other international and nongovernmental bodies, as appropriate.  -  - Ensure independence and competence of the reviews.) | The CPP is the body in charge of the protection of individuals, the evaluation of the relevance of the research, the benefit-risk  and patient compensation. The CPP gives its opinion on the conditions of validity of the research protection of participants, the methods of recruitment, information and recruitment, information and compensation, the relevance of the research of the research, the qualifications of the investigators and the evaluation of of benefits and risks. There are 40 CPPs spread over 7 inter-regions. The composition of the CPP comprises 14 full members divided into two colleges in accordance with the provisions of article **L. 1114-1**. The first college is composed of four persons with a qualification and in-depth experience in research involving the involving the human person (including at least two physicians and one person and one person qualified by virtue of his or her competence in the field of biostatistics or epidemiology), a general practitioner a hospital pharmacist and a nurse. The second college is composed of a person qualified in ethical issues, a psychologist, a social worker  social worker, two persons qualified by their competence in legal matters  two persons qualified by their competence in legal matters and two representatives of associations. Each committee includes among its members a person qualified in data protection. Alternate members equal in number to the number of full members are members are appointed for each category under the same conditions. conditions. It is also composed of pediatricians and specialists for studies on children or on persons of full age who are unable to give their incapable of giving their consent. The opinion is given within 45 days. The National Commission for Research Involving the Human Person coordinates and coordinates and harmonizes the functioning of the different CPP.  CS for independance of the CPP formulated in **Art. L1123-1** “The fact that a member of the National Commission on Research Involving the Human Person takes part in the work or deliberations of the National Commission when he or she has a direct or indirect interest in the case under examination is punishable by five years' imprisonment and a fine of €75,000” | **Article 4**  Prior authorisation  - The ethical review shall be performed by an ethics committee in accordance with the law of the Member State concerned. The review by the ethics committee may encompass aspects addressed in Part I of the assessment report for the authorisation of a clinical trial as referred to in Article 6 and in Part II of that assessment report as referred to in Article 7 as appropriate for each Member State concerned.  **Article 9:**  1) Member States shall ensure that the persons validating and assessing the application do not have conflicts of interest, are independent of the sponsor, of the clinical trial site and the investigators involved and of persons financing the clinical trial, as well as free of any other undue influence.  In order to guarantee independence and transparency, the Member States shall ensure that persons admitting and assessing the application as regards the aspects addressed in Parts I and II of the assessment report have no financial or personal interests which could affect their impartiality. These persons shall make an annual declaration of their financial interests.  2. Member States shall ensure that the assessment is done jointly by a reasonable number of persons who collectively have the necessary qualifications and experience.  3. At least one layperson shall participate in the assessment. | - Yes, IRB, Egyptian Drug Authority and Supreme  Council    -   No.     -Institutional Committee for the Review of Medical Research Ethics (Institutional Committee (Intuitional Review Board): A group of persons with medical and non-medical specialties, who reviews research plans (protocols) and apply ethical principles that must be followed in this regard. This committee is located in the research facility, and must be registered with the Supreme Council. It is referred to in this law as the (Intuitional Review Board). Art1; 24    - A committee called "Institutional Review Board" shall be established within each research facility as decreed by the relevant authority in such facility. This committee must be registered in the Supreme Council. This committee is authorized to:  1- Protect the rights, safety and health of the research subjects.  2- Review the research protocols submitted to it and ensure that protocols contain all the papers, approvals and documents necessary in this regard, according to each case.  3- Issue a decision to approve conducting of or renew the medical research, determining its duration to not exceed one year, and following it up until it is terminated or completed.  4- The Principal Investigator and sponsor of the research, if applicable, to ensure that the medical research is properly carried out and that standards of good clinical practice are applied. The IRB shall notify the Supreme Council of all research submitted to it, provided that the notification includes the medical research protocol and all documents and data related to it, as well as all measures taken by the committee in this regard. All this is in accordance to the executive regulations of **law art 8.**  **Chap 2 article 4**: IRB  **Chap 3 artile 1 to 4** about setting up protocols | 6 § Research referred to in Sections 3-5 may only be carried out if it has been approved by an ethics review. The salient part in Sections 3-5 is section 4, where it is stated that the law shall be applied to research which  1. involves a physical intervention on a research subject,  2. is carried out according to a method that aims to affect the research subject physically or psychologically or which involves an obvious risk of harming the research subject physically or psychologically,  3. refers to studies on biological material that has been taken from a living person and can  be traced to that person,  4. involves a physical intervention on a deceased person, or  5. refers to studies on biological material that has been taken for medical purposes from a deceased person and can be traced to that person.  **6 §** A principal investigator (research institution?) must take measures to prevent research in his own activities from being carried out in violation of the *law.*  **25 §** The Ethics Review Authority shall be divided into operational regions. Each operating region must have one or more departments. A department must examine cases in specific research areas. A department shall consist of a chairman and fifteen other members. Of the other members, ten must have scientific competence and five must represent public interests. Substitutes may be appointed for the members. The chairman and substitute for the chairman must be or have been ordinary judges. The chairman and deputy for the chairman must be appointed by the government. Other members and substitutes must be appointed by the Ethics Review Authority. All members and substitutes must be appointed for a fixed period (…at least one of the members who represent public interests represents one or more patient organizations).  **SFS 2018:1091** Act with supplementary provisions on ethical review to the EU regulation on clinical trials of human medicinal products Ethical review of the application for permission for clinical drug trials § 2 The ethical review must be carried out by the Ethics Review Authority. |
| **Informed consent**  Provision of information to subjects about purpose of the research, its procedures, potential risks, benefits, and alternatives, so that the individual understands this information and can make a voluntary decision whether to  enroll and continue to participate.  (-  Involve the community in establishing recruitment procedures and incentives.  - Disclose information in culturally and linguistically appropriate formats.  - Implement supplementary community and familial consent procedures where culturally appropriate.  - Obtain consent in culturally and linguistically appropriate formats.  Ensure the freedom to refuse or withdraw ) | **Article L1121-2**  The information and consent of the patient participating in the research are essential elements of good clinical research practices. For category 1 research, the information must be individual and consent must be free, informed and collected in writing. For category 2 research, the information must be individual, but a waiver for collective information can be requested from the CPP. Consent is free, informed and "express". It must be written for category 1 studies and may be oral for category 2 studies (but must be recorded in the medical file). For category 3 research and for studies on data collected in the normal course of normal care, the rule is that the patient must not object. For this, the patient must be informed in order to be able to refuse or to express his opposition at any time. The information must contain the identity and contact details of the data controller and the data protection officer, the purpose of the data processing, and the name of the person responsible for the data. (presentation of the legal basis of the data processing, the nature of the information that will be used in the research the recipients or categories of recipients of the data, the rights of access, rectification, opposition, deletion, limitation of data to the data processing, the modalities of exercise of these rights, the optional nature of participation, if necessary the transfer of personal data outside the European Union, the reference to appropriate guarantees, and finally the duration of personal data or the criteria used to determine this period.)  **Article L1121-14** No research may be carried out on a deceased, brain-dead person without his or her consent expressed during his or her lifetime or through the testimony of his or her family.  **Chapter II: Informing and obtaining the consent of persons undergoing research involving the human person (Articles L1122-1 to L1122-2).**  **Chapitre V : Dispositions particulières applicables aux investigations cliniques de dispositifs mentionnés à l'article premier du règlement (UE) 2017/745 du Parlement européen et du Conseil du 5 avril 2017 (Articles L1125-1 à L1125-31)** | **Article 7**   1) a) compliance with the requirements for informed consent as set out in **Chapter V**;  **Chapter 5 Article 29**  **Informed consent** 1. Informed consent shall be written, dated and signed by the person performing the interview referred to in point (c) of paragraph 2, and by the subject or, where the subject is not able to give informed consent, his or her legally designated representative after having been duly informed in accordance with paragraph 2. Where the subject is unable to write, consent may be given and recorded through appropriate alter­ native means in the presence of at least one impartial witness. In that case, the witness shall sign and date the informed consent document. The subject or, where the subject is not able to give informed consent, his or her legally designated representative shall be provided with a copy of the document (or the record) by which informed consent has been given. The informed consent shall be documented. Adequate time shall be given for the subject or his or her legally designated representative to consider his or her decision to participate in the clinical trial. 2. Information given to the subject or, where the subject is not able to give informed consent, his or her legally designated representative for the purposes of obtaining his or her informed consent shall: (a)  enable the subject or his or her legally designated representative to understand: (i)  the nature, objectives, benefits, implications, risks and incon­veniences of the clinical trial (ii)  the subject's rights and guarantees regarding his or her protection, in particular his or her right to refuse to participate and the right to withdraw from the clinical trial at any time without any resulting detriment and without having to provide any justification; (iii)  the conditions under which the clinical trial is to be conducted, including the expected duration of the subject's participation in the clinical trial; and (iv)  the possible treatment alternatives, including the follow-up measures if the participation of the subject in the clinical trial is discontinued; (b)  be kept comprehensive, concise, clear, relevant, and understandable to a layperson;  (c)  be provided in a prior interview with a member of the investigating team who is appropriately qualified according to the law of the Member State concerned; (d)  include information about the applicable damage compensation system referred to in **Article 76(1**); and (e) include the EU trial number and information about the availability of the clinical trial results in accordance with paragraph 6. 3. The information referred to in paragraph 2 shall be prepared in writing and be available to the subject or, where the subject is not able to give informed consent, his or her legally designated representative.4. In the interview referred to in point (c) of paragraph 2, special attention shall be paid to the information needs of specific patient populations and of individual subjects, as well as to the methods used to give the information. 5. In the interview referred to in point (c) of paragraph 2, it shall be verified that the subject has understood the information. 6. The subject shall be informed that the summary of the results of the clinical trial and a summary presented in terms understandable to a layperson will be made available in the EU database, referred to in **Article 81** (the ‘EU database’), pursuant to **Article 37(4),** irrespective of the outcome of the clinical trial, and, to the extent possible, when the summaries become available. 7. This Regulation is without prejudice to national law requiring that both the signature of the incapacitated person and the signature of his or her legally designated representative may be required on the informed consent form. 8. This Regulation is without prejudice to national law requiring that, in addition to the informed consent given by the legally designated representative, a minor who is capable of forming an opinion and assessing the information given to him or her, shall also assent in order to participate in a clinical trial. **Article 30** **Informed consent in cluster trials** 1. Where a clinical trial is to be conducted exclusively in one Member State, that Member State may, without prejudice to Article 35, and by way of derogation from points (b), (c), and (g) of Article 28(1), Article 29(1), point (c) of Article 29(2), Article 29(3), (4) and (5), points (a), (b) and (c) of Article 31(1) and points (a), (b) and (c) of Article 32(1), allow the investigator to obtain informed consent by the simplified means set out in paragraph 2 of this Article, provided that all of the conditions set out in paragraph 3 of this Article are fulfilled. 2. For clinical trials that fulfil the conditions set out in paragraph 3, informed consent shall be deemed to have been obtained if:  (a)  the information required under points (a), (b), (d) and (e) of Article 29(2) is given, in accordance with what is laid down in the protocol, prior to the inclusion of the subject in the clinical trial, and this information makes clear, in particular, that the subject can refuse to participate in, or withdraw at any time from, the clinical trial without any resulting detriment; and (b)  the potential subject, after being informed, does not object to participating in the clinical trial. 3. Informed consent may be obtained by the simplified means set out in paragraph 2, if all the following conditions are fulfilled: (a)  the simplified means for obtaining informed consent do not contradict national law in the Member State concerned; (b)  the methodology of the clinical trial requires that groups of subjects rather than individual subjects are allocated to receive different investigational medicinal products in a clinical trial; (c)  the clinical trial is a low-intervention clinical trial and the investigational medicinal products are used in accordance with the terms of the marketing authorisation;(d)  there are no interventions other than the standard treatment of the subjects concerned; (e)  the protocol justifies the reasons for obtaining informed consent with simplified means and describes the scope of information provided to the subjects, as well as the ways of providing information. 4. The investigator shall document all refusals and withdrawals and shall ensure that no data for the clinical trial are collected from subjects that refuse to participate in or have withdrawn from the clinical trial. | Informed consent: the written expression based on complete voluntary freewill of the person with full legal capacity, and it includes his explicit consent as a signature and a fingerprint to participate in clinical medical research, after all aspects of the research are explained to him, and in particular the potential effects or harms that may impact his/her decision to participate. This approval is issued by his/her legal representative in the cases referred to in the provisions of this law”. Art 1; 21    The exception of obtaining approval (informed consent) from the research subject; the situations that necessitate proper (scientific) implementation of the medical research as explained in Egyptian laws and rules governing data handling and confidentiality. But in a way that does not disrupt investigative officials or a court order requesting the disclosure of this data as seen necessary and is required by investigation or persecuting proceedings[MOU1] .  All this is as detailed in the executive regulations of this law. Art 12; 3  **Chap.5 arti 12 § 3**  It may not be permitted to conduct (limit) medical research to a specific group of people or vulnerable populations unless the research is necessary and addresses their specific diseases, and providing the scientific and ethical justifications for their enrollment, and provided that informed consent is obtained from each of them. If the medical research is to be conducted on one of the vulnerable populations, the consent of the two parents, or whoever has the legal guardianship in the event of the death of one or both of the parents, or the legal representative must be obtained, in accordance with the rules and procedures specified by the executive regulations of this **law Art 3**  **Chap 7 art 17 § 2**: obtaining IC is mandatory  **Chap 10, art 23 §2** Informed consent for data usage for further research | **!6 §** The research subject must be informed about  - the overall plan for the research,  - the purpose of the research,  - the methods that will be used,  - the consequences and risks that the research may entail,  - who is the principal investigator,  - that participation in the research is voluntary, and  - the research subject's right to withdraw their participation at any time.  **14 §** If a research subject is in a dependent relationship with the principal investigator or a researcher, or if the research subject can be assumed to have particular difficulties in asserting his right, issues of information and consent must be given special attention during the ethics review.  **17 §** Research may only be carried out if the research subject has consented to the research concerning him or her. Consent only applies if the research subject has previously received information about the research according to § 16. Consent must be voluntary, explicit and specific to the research. The consent must be documented.  **18 §** If the research subject has turned 15 but not 18 and realizes what the research implies for him or her, he or she must be informed about and consent to the research.  In other cases, when the research subject has not reached the age of 18, the guardians must be informed about and consent to the research. The research subject himself must, however, be informed about the research as much as possible. Despite the consent of the guardians, the research may not be carried out if a research subject under the age of 15 realizes what it implies for him or her and objects to it being carried out.  **20 §** Research may be carried out without consent, if illness, mental disorder, weakened state of health or any other similar condition of the research subject prevents his or her opinion being obtained. However, the research may only be carried out during those prerequisites specified in §§ 21 and 22.  **21 §** Research concerning a research subject as stated in § 20 may be carried out if 1. the research can be expected to provide knowledge that is not possible to obtain through research with consent, and 2. the research can be expected to lead to direct benefit for the research subject. Even if the condition in paragraph 2 is not met, the research may be carried out if 1. the purpose is to contribute to a result that may be of benefit to the research subject or someone else suffering from the same or similar disease or disorder, and 2. the research involves an insignificant risk of injury and an insignificant discomfort for the research subject.  **22 §** A research subject referred to in § 20 must be informed personally about the research as much as possible. Consultation must take place with the research subject's next of kin. Consultation must also take place with a trustee or administrator according to ch. 11. of the parental code, if the issue is part of his duties. The research may not be carried out if the research subject expresses in any form that they do not want to participate or if any of those who have been consulted opposes the execution.  **All the sections in the law from 13, 14, 15, 16, 17, 18, 19, 20 ,21 and  22** are relevant to this section |
| **Respect for recruited participants and study communities**    Respect for subjects by  (1) permitting withdrawal from the  research;  (2) protecting privacy through Confidentiality;  (3) informing subjects of newly discovered risks or benefits;  (4) informing subjects of results of clinical research;  (5) maintaining welfare of subjects  (-Develop and implement procedures to protect the confidentiality of recruited and enrolled participants.  - Ensure that participants know they can withdraw without penalty.  - Provide enrolled participants with information that arises in the course of the research study. - Monitor and develop interventions for medical conditions, including research-related injuries, for enrolled participants at least as good as existing local norms.  - Inform participants and the study community of the results of the research.) | The French law defines three important vulnerable groups in all:  1. Persons deprived of their freedom by a judicial or administrative decision;   2. Minors, adults under guardianship, persons in medical or social establishments and patients in emergency situations, and  3. Pregnant or nursing women. The most restrictive regulations are in regard to the groups of persons deprived of their liberty by a judicial or administrative decision, in particular, to prisoners. The law allows no research without direct benefit to this group and this without exception (art L 209–5). There are also strict criteria governing therapeutic trials in regard to persons deprived of their liberty. The legitimacy of therapeutic research is, in such cases, bound explicitly to a “greater benefit” (bénéfice majeur). In this manner the danger of an improper influencing of prison inmates is prevented.  **Information and patient consent** According to key elements of the Nuremberg code, respecting the human subject requires providing them with information and obtaining their consent – the modalities of which vary according to the type of study.  **Information** For the first two categories of research, the information provided must be very detailed and summarized on a written document delivered by the promoter of the study, a doctor or a qualified person who represents them (**art. L.1122-1 PHC**). For research not entailing listed risks, the information to be delivered may be more laconic (**art. L.1122-1 HPC**). In practice, this led to the use of simplified information notes. Information must also include the protection of personal data, pursuant to chapter III GDPR. For studies falling outside of the Jardé law, the information of participants remains mandatory, since patient information laws (Law 2002-303 known as Koushner law) and the use and protection of personal data (GDPR) are applicable. It is mandatory to plan the adaptation of the documents designed for the vulnerable (protected adults or minors) and foreigners (note in their mother tongue). **Consent:** As mentioned in the Nuremberg Code, consent is indispensable to carry out any research. Methods to obtain it may vary, depending on the type of research and risks and constraints for each. To abstain from obtaining it is severely punished. Thus, carrying out or having clinical trials falling in categories 1 or 2 carried out without collecting the person's consent beforehand is punished with 3 years of imprisonment and a fine of 45,000€. The same sanctions are applicable to category 3 researches where the person objected to participate (art L1126-1 PHC). **Concerning interventional researches (category 1)**: a free, informed and written consent is required (art. L.1122-1-1 PHC), as well as the signature of a form containing the list of mandatory information et indicating their rights to the person participating and especially their right to interrupt their participation at any stage of the care provided for them without being liable. A little precision must be added in case the person cannot write. Their consent can only be verified by the designated person of trust, a family member or close relative (**art. L. 1122-1-1 PHC**). This requirement must be mentioned in the consent form. **Concerning interventional researches entailing minimal risks and constraints (category 2)** (**art. L.1122-1-1, §2, PHC**), a free, informed and express consent is required. Written consent is not mandatory, but proof of consent often makes a written document indispensable. In this category, it is now possible to prohibit simultaneous participation to another study (which used to be only applicable for category 1 studies before). | **Article 28**  **General rules**  1. A clinical trial may be conducted only where all of the following conditions are met:  (a)  the anticipated benefits to the subjects or to public health justify the foreseeable risks and inconveniences and compliance with this condition is constantly monitored; (b)  the subjects, or where a subject is not able to give informed consent, his or her legally designated representative, have been informed in accordance with **Article 29**(2) to (c)  the subjects, or where a subject is not able to give informed consent, his or her legally designated representative, have given informed consent in accordance with **Article 29**(1), (7) and (8); (d)  the rights of the subjects to physical and mental integrity, to privacy and to the protection of the data concerning them in accordance with Directive 95/46/EC are safeguarded; (e)  the clinical trial has been designed to involve as little pain, discomfort, fear and any other foreseeable risk as possible for the subjects and both the risk threshold and the degree of distress are specifically defined in the protocol and constantly monitored; (f)  the medical care provided to the subjects is the responsibility of an appropriately qualified medical doctor or, where appropriate, a qualified dental practitioner; (g)  the subject or, where the subject is not able to give informed consent, his or her legally designated representative has been provided with the contact details of an entity where further information can be received in case of need; (h)  no undue influence, including that of a financial nature, is exerted on subjects to participate in the clinical trial. **Chapter 5, Article 28**  3. Any subject, or, where the subject is not able to give informed consent, his or her legally designated representative, may, without any resulting detriment and without having to provide any justification, withdraw from the clinical trial at any time by revoking his or her informed consent. Without prejudice to Directive 95/46/EC, the withdrawal of the informed consent shall not affect the activities already carried out and the use of data obtained based on informed consent before its withdrawal. | “Not to reveal his identity [MOU1] or any of his data except after provided with scientific justifications as approved by the Institutional Review Board and authorized by the Supreme Council, and with the written approval of the research subject or his legal representative.” **Art 12; 2.**  “Make available all information, data and reports related to the medical research [MOU2] during or after the completion of the research to the respective IRB, the Supreme Council, the General Intelligence Agency and the Egyptian Drug Authority, for the purpose of auditing and reviewing”  Art, 15; 2.Chap 5  Avoid publishing or advertising in newspapers or the media about any information, or data or reports of the medical research, except after its completion and obtaining a written approval of the respective IRB and the Supreme Council, and the written consent of the participating research subjects in case of any disclosure of any statements or data [MOU3] related to them Art 15;3    -“1- The right to withdraw from the medical study whenever he wishes and without being obligated to give reasons for withdrawal, provided that the Principal Investigator informs him of the medical harm resulting from his withdrawal. ”**Art 12 (1),** subject’s rights    -Informing the research subject of any modifications that will be made to the research plan that may affect his safety, as well as informing him of any unforeseen risks that may occur to him or to other participating subjects during the implementation of medical research. **art 18:5**    8- Immediately informing the research subjects participating in the medical research, and promptly, of any modifications made to it (medical research) and of any results that may negatively affect the safety of the participating subjects, as well as of the un-anticipated serious  side effects of the medical research  9 - Commitment to conclude/make an insurance contract for research subjects participating in medical research with one of the insurance companies accredited in the Arab Republic of Egypt, with the aim of facing/handling any damages that may result from participating in the medical research. The contract referred to in this clause shall cover the period of medical research and the period of follow-up, so that it is valid for a year following the completion of the medical research. The value of this insurance is approved by the Supreme Council.  10 - Compensating and treating research subjects in the case of injury related to medical research.  11- Completing treatment of the participating research subjects when it is demonstrated/established their need for it even after the end of the medical research. All this is as specified by the executive regulations of this law**. Art 20:9, 10** (Sponsor  It is prohibited to induce research subject to participate in medical research by offering them rewards or cash or other benefits. Undue inducement . **Art 14** | **1§** The purpose of the law is to protect the individual and respect for human dignity in research.  **7 §** Research may only be approved if it can be carried out with respect for human dignity. *(listed as the first starting point of ethics review)*  **19 §** Consent may be withdrawn at any time with immediate effect. However, the data that have been collected before that can be used in the research.  **8 §** :Human rights and fundamental freedoms must always be taken into account during the ethical review, while account must be taken of the interest in new knowledge being developed through research. People's welfare must be given priority over the needs of society and science. |
| **Extras/Miscellaneous** | <https://www.legifrance.gouv.fr/codes/section_lc/LEGITEXT000006072665/LEGISCTA000006154978/>  Regulation on the financial issues around clinical trials is evaluated in the CS (**Art L1121-10, L1121-11**, with specific emphasis in Psychiatric care and midwifery)and products need to be free for participants (**L1121-13-1 updated in 2016**).  The place where the study take place need to be able to guaranty the security of the participants (Article L1121-13) look restrictive to hospitals ?  Specific cases like research on a dead person are regulated by Penal Code (**Article 225-17** even if the CS had the L1121-14 article refereeing to it):The purpose of psychological research, as well as its methodology and duration, may be the subject of only brief prior information if the research does not present any foreseeable serious risk. Full information on this research is provided to the persons who have undergone it. The project mentioned in article L. 1123-6 mentions the nature of the prior information transmitted to the persons undergoing the research.  Regarding informed consent, **CS L1121-1-1** updated in 2016 specifically says “No research mentioned in 2**° of Article L. 1121-1** may be carried out on a person without his/her free, informed and express consent.” that may contradict some rules for collective consent ?  **Art L1123-10:** rule to report side effects. Last chapters: Chapter V: Special provisions applicable to clinical investigations of devices mentioned in Article 1 of Regulation (EU) 2017/745 of the European Parliament and of the Council of 5 April 2017 (Articles L1125-1 to L1125-31) Chapter VI: Special provisions applicable to studies of the performance of devices mentioned in Article 1 of Regulation (EU) 2017/746 of the European Parliament and of the Council of 5 April 2017 (Articles L1126-1 to L1126-29) Chapter VII: Special provisions for certain research (Articles L1127-1 to L1127-4) Chapter VIII: Criminal provisions (Articles L1128-1 to L1128-12) | **Article 35 Clinical trials in emergency situations** 1**. By way of derogation from points (b) and (c) of Article 28(1), from points (a) and (b) of Article 31(1) and from points (a) and (b) of Article 32(1),** informed consent to participate in a clinical trial may be obtained, and information on the clinical trial may be given, after the decision to include the subject in the clinical trial, provided that this decision is taken at the time of the first intervention on the subject, in accordance with the protocol for that clinical tria**l"** and that all of the following conditions are fulfilled:(a)  due to the urgency of the situation, caused by a sudden life- threatening or other sudden serious medical condition, the subject is unable to provide prior informed consent and to receive prior information on the clinical trial;(b)  there are scientific grounds to expect that participation of the subject in the clinical trial will have the potential to produce a direct clinically relevant benefit for the subject resulting in a measurable health-related improvement alleviating the suffering and/or improving the health of the subject, or in the diagnosis of its condition; (c)  it is not possible within the therapeutic window to supply all prior information to and obtain prior informed consent from his or her legally designated representative; (d)  the investigator certifies that he or she is not aware of any objections to participate in the clinical trial previously expressed by the subject; (e)  the clinical trial relates directly to the subject's medical condition because of which it is not possible within the therapeutic window to obtain prior informed consent from the subject or from his or her legally designated representative and to supply prior information, and the clinical trial is of such a nature that it may be conducted exclusively in emergency situations; (f)  the clinical trial poses a minimal risk to, and imposes a minimal burden on, the subject in comparison with the standard treatment of the subject's condition.2. Following an intervention pursuant to paragraph 1, informed consent in accordance with **Article 29** shall be sought to continue the participation of the subject in the clinical trial, and information on the clinical trial shall be given, in accordance with the following requirements: (a)  regarding incapacitated subjects and minors, the informed consent shall be sought by the investigator from his or her legally designated representative without undue delay and the information referred to in **Article 29(2)** shall be given as soon as possible to the subject and to his or her legally designated representative; (b)  regarding other subjects, the informed consent shall be sought by the investigator without undue delay from the subject or his or her legally designated representative, whichever is sooner and the information referred to in Article 29(2) shall be given as soon as possible to the the subject or his or her legally designated representative, whichever is sooner. For the purposes of point (b), where informed consent has been obtained from the legally designated representative, informed consent to continue the participation in the clinical trial shall be obtained from the subject as soon as he or she is capable of giving informed consent. 3. If the subject or, where applicable, his or her legally designated representative does not give consent, he or she shall be informed of the right to object to the use of data obtained from the clinical trial. | **Executive regulations ch 5 article 22:**  Researchers declaration of conflict of interest.  Executive reg Ch 7 art 29: committee that reviews appeals | **§ 40** The government or the authority that the government determines may prescribe exemptions from the requirement for approval during ethics review for research or the processing of personal data, where it is clear that the research does not entail any appreciable risk to an individual's health or safety or to an infringement of an individual's personal integrity  **Penalty Section 38** Anyone who intentionally violates Section 6, first paragraph or a condition that has been announced pursuant to Section 6, first paragraph, is sentenced to a fine or imprisonment for a maximum of two years. The same applies to anyone who intentionally fails to take the measures that could reasonably be required according to Section 6, second paragraph, if research in their own activities is carried out in violation of Section 6, first paragraph or a condition that has been announced in support of Section 6, first paragraph. If the act according to the first or second paragraph is committed through gross negligence, the sentence is a fine or imprisonment for a maximum of six months. In minor cases, there is no liability. Anyone who has violated a penalty order according to section 35 may not be held liable for an act covered by the order. **Law (2019:1144)** |
